# Dietary intakes, food behaviours and health indicators among Métis youth in Manitoba, Canada

**DOI:** 10.1017/S1368980025000151

**Published:** 2025-02-04

**Authors:** Chantal Perchotte, Olena Kloss, Joyce Slater, Alan Katz, Bhanu Pilli, Aynslie Hinds, Marcelo L Urquia, Julianne Sanguins, Chris Green, Jaime Cidro, Dan Chateau, Nathan Nickel, Thomas Falkenberg

**Affiliations:** 1 Manitoba Métis Federation, Winnipeg, MB, Canada; 2 Department of Food and Human Nutritional Sciences, University of Manitoba, Winnipeg, MB, Canada; 3 Department of Community Health Sciences, Max Rady College of Medicine, University of Manitoba, Winnipeg, MB, Canada; 4 Department of Family Medicine, Max Rady College of Medicine, University of Manitoba, Winnipeg, MB, Canada; 5 Department of Psychology, University of Winnipeg, Winnipeg, MB, Canada; 6 Winnipeg Regional Health Authority, Winnipeg, MB, Canada; 7 Department of Anthropology, University of Winnipeg, Winnipeg, MB, Canada; 8 Research School of Population Health, Australian National University, Canberra, Australia; 9 Faculty of Education, University of Manitoba, Winnipeg, MB, Canada

**Keywords:** Nutrition, Food security, Indigenous, Adolescent, Métis, Canada

## Abstract

**Objective:**

Poor diets and food insecurity during adolescence can have long-lasting effects, and Métis youth may be at higher risk. This study, as part of the Food and Nutrition Security for Manitoba Youth study, examines dietary intakes, food behaviours and health indicators of Métis compared with non-Métis youth.

**Design:**

This observational cross-sectional study involved a cohort of adolescents who completed a self-administered web-based survey on demographics, dietary intake (24-h recall), food behaviours, food security and select health indicators.

**Setting:**

Manitoba, Canada

**Participants:**

Participants included 1587 Manitoba grade nine students, with 135 (8·5 %) self-identifying as Métis, a distinct Indigenous nation living in Canada.

**Results:**

Median intake of sugar was significantly higher in Métis (89·2 g) compared with non-Métis (76·3 g) participants. Percent energy intake of saturated fat was also significantly higher in Métis (12·4 %) than non-Métis (11·6 %) participants. Median intakes of grain products and meat and alternatives servings were significantly lower among Métis than non-Métis (6·0 *v*. 7·0 and 1·8 *v*. 2·0, respectively) participants. Intake of other foods was significantly higher in Métis (4·0) than non-Métis (3·0). Significantly more Métis participants were food insecure (33·1 %) compared with non-Métis participants (19·1 %). Significantly more Métis participants ate family dinners and breakfast less often than non-Métis participants and had lower self-reported health. Significantly more Métis participants had a BMI classified as obese compared with non-Métis participants (12·6 % *v*. 7·1 %).

**Conclusions:**

The dietary intakes observed in this study, both among Métis and non-Métis youth, are concerning. Many have dietary patterns that put them at risk for developing health issues in the future.

Many physiological changes occur during adolescence, including those related to puberty, growth spurts and neurological development^(1)^. Due to the rapid pace of physical development during this period, energy and nutrient needs are high^(2)^. Certain nutrients are key to supporting growth during this life stage. Ca needs are highest during adolescence to support many functions throughout the body, including bone growth^(3)^. Bone development is also sensitive to adequate intake of nutrients such as vitamins D and K, Fe and protein^(2)^. Fe requirements progressively increase throughout childhood and adolescence to support increased blood production, and Fe needs increase further for females when menstruation begins^(3)^. While this is an important time for adequate nutrition, adolescents in Canada have low dietary intakes of many nutrients, including vitamins A, C and D, Ca, Zn and fibre^(3)^.

Concerns around adequate dietary intake are exacerbated for adolescents facing food insecurity and other social and health disparities, which are disproportionately experienced by Indigenous populations in Canada. Colonial institutions and practices throughout Canada’s history have differentially impacted the three Indigenous groups recognised in the 1982 Constitution Act – First Nations, Métis and Inuit. First Nations peoples have historically been the subjects of the Indian Act, which continues to legally define and impact First Nations peoples. In contrast, Métis have suffered a lack of recognition as an Indigenous people of Canada for most of their history. Descendants of early 17th-century relationships between North American Indians and European settlers^(4)^, the Métis coalesced into a distinct nation in Manitoba by the early 18th century. The Red River Settlement, now known as Winnipeg, is the birthplace of the Métis Nation and the heart of the Métis Homeland. The Red River Métis people share a distinct identity and common history with roots in the western prairies centred in the Red River Valley^(5)^. The Red River Métis is made up of Métis Citizens and settlements and is defined by a common ancestry, identity, culture, kinship and history^(5)^. Until the amendment to the Canadian Constitution in 1982 naming Métis as one of the three groups of Indigenous peoples, Métis were recognised neither as Indigenous nor as fully European or Canadian. This has resulted in exclusion from treaties, land settlements and until recently, Indigenous hunting and gathering rights.

The enduring effects of colonial policies and practices are evident in ongoing disparities between Métis and non-Métis in various social determinants of health and health outcomes, including those related to nutrition. When compared with all other Manitobans, Métis children are over twice as likely to be in families receiving provincial income assistance and twice as likely to receive provincial income assistance as young adults (18–19 years)^(6)^. This contributes to food insecurity observed in both youth and Indigenous populations. In 2022, one in four children under the age of 18 in Canada lived in households experiencing food insecurity^(7)^. Additionally, the second highest percentage of individuals living in food-insecure households in Canada is off-reserve Indigenous peoples at 33·4 % in comparison with people identifying as white at 15·3 %^(7)^. These alarming statistics suggest Indigenous youth are particularly vulnerable to circumstances of food insecurity, which limit access to healthy food and negatively impact dietary intake^(7)^. Experiencing food insecurity, particularly during the critical development stage of adolescence, may negatively impact health, including associations with reduced cognitive function, poor physical health and chronic conditions such as CVD and diabetes^(7)^. These implications of food insecurity are particularly concerning within the context of the Métis population, who are already at an increased risk of adverse health outcomes. The Métis population in Manitoba has a significantly higher prevalence of diabetes and CHD compared with all other Manitobans (11·8 % *v*. 8·8 % and 12·2 % *v*. 8·7 %, respectively)^(6)^. When compared with all other Manitobans, the Métis population has significantly higher rates of premature mortality at 4·0 deaths per 1000 people aged 0–74 compared with 3·3 per 1000 for all other Manitobans^(6)^.

Collectively, the colonial policies and practices discussed impact adolescents’ dietary patterns, frequency of meals and the amount of food intake, resulting in inadequate nutrient supply, which leads to aberrations in critical physiological processes taking place during maturation and development. Notably, the Métis population has a greater proportion of youth (0–19 years) when compared with other Manitobans^(6)^. Poor nutrition during this life stage can also increase the risk of developing non-communicable disease (NCD) later in life^(3)^. Thus, there is a need for nutrition policy and intervention to reduce the nutritional risks associated with the development of NCD in Métis youth.

This study, as part of the larger Food and Nutrition Security for Manitoba Youth (FANS) study, analysed data collected from grade nine students attending Manitoba schools to describe the dietary intakes, food behaviours and health indicators of Métis youth compared with those of non-Métis youth.

## Methods

### Study design

An observational cross-sectional study design was employed with survey data collected from grade nine students attending Manitoba public schools during the 2018–2019 academic year. Written consent was obtained from a parent/guardian of each student and students provided their individual assent at the beginning of the online survey.

Students completed the online survey during a regular school day. A trained research assistant was present to respond to questions and help with any technical issues. The survey involved four components, including (1) demographic characteristics, (2) questions related to experiences of food insecurity, (3) a 24-h diet recall and (4) eating behaviour and self-reported health questions. Each student was provided with a unique number for anonymisation, and collected data were stored on a secure server to which only authorised study personnel had access.

Ethics approval for this study was obtained from the Joint Faculty Research Ethics Board at the University of Manitoba (protocol HS2166 J2018:040), and all experimental methods were performed in accordance with the relevant guidelines and regulations. Detailed methods for the FANS study have been published elsewhere^(8)^

#### Settings

The FANS study took place in Manitoba, Canada, a province with a population of 1 342 153^(9)^. The city of Winnipeg is the largest urban centre in the province, having a population of 749 607^(9)^.

### Participants

A stratified two-stage method was used to recruit grade nine students attending public schools in Manitoba. Grade nine was selected as students have the independence to complete the self-administered survey and are at a critical developmental stage during which adequate nutrition is imperative.

The largest eighteen of the thirty-seven school divisions throughout Manitoba were approached, with fourteen agreeing to participate in the study. Nineteen school divisions were excluded due to few grade 9 students and/or data collection cost restrictions. Among the fourteen agreeing school divisions, schools with classes of ten or more grade 9 students were invited to participate. School divisions and schools were classified into urban, northern and rural regions: school divisions in the Winnipeg Health Region are urban; divisions in the Northern Health Region are northern, and the remaining divisions are considered rural. Thirty-seven of sixty-two eligible schools participated: twenty-four in urban school divisions, five in northern and eight in rural divisions. A detailed description of the FANS study design and rationale is reported elsewhere^(8)^.

Indigenous ancestry was self-reported (First Nations, Métis, Inuit, Don’t Know). Questions surrounding Indigeneity were developed with Indigenous academic and community partners.

### Measures

Dietary intakes, food behaviours and health indicators were obtained using the Waterloo Eating Behaviour Questionnaire (WEB-Q), a validated online tool for measuring the food and nutrient intake of adolescents using a 24-h dietary recall and FFQ^(10)^. Dietary intake was assessed through a 24-h recall module. Students selected options from a list of approximately 800 food items, which were categorised into meals and snacks. Students chose food and beverages and portion sizes based on pictures and associated text on the screen. In addition to the 24-h recall, participants provided responses on the consumption frequency of sugar-sweetened, caffeinated and high-protein beverages. Using the online WEB-Q, food behaviour was assessed via questions about the frequency of meal consumption, meals consumed with family members and food purchasing habits. Health indicators included questions about eating-related weight control and sleep behaviours. Self-reported health and life satisfaction measures were included. BMI was calculated using self-reported height and weight and classified using WHO z-scores.

Food security was assessed using the Child Food Security Survey Module validated for youth over 12 years of age. The module consists of nine questions focused on access to food, concerns about food availability, modified eating behaviours and hunger levels within the past 12 months.

### Statistical analysis

Study data were analysed using SAS (version 9.4, SAS Institute Inc. 2023) (variable derivation) and SPSS (version 27, IBM Corp., 2020) (tables and statistical outputs) statistical software packages and Microsoft Excel (95 % CI). Mean and median nutrient and food group intakes were calculated with corresponding measures of variability. Differences by group in median nutrient and food group intakes were assessed using the Mann–Whitney *U* test. *χ* ^2^ tests were performed for comparing the percentage of Métis and non-Métis participants not meeting key nutrient and food group recommendations, and for food security, food behaviour and health indicator variables. Significant differences in BMI categories were determined using the z-score test for two population proportions. Statistical significance was accepted at *P*< 0·05. N-values vary slightly in the tables because some of the students did not answer all the questions.

## Results

### Participant characteristics

Table 1 presents the age and sex of the study participants. There were almost even numbers of males and females in the Métis and non-Métis groups; however, there were slightly more 15-year-olds compared with 14-year-olds in the Métis group. Additional information on the geographic location of study participants has been published elsewhere^(11)^.

**Table 1.** Characteristics of study participants

	Métis (*n* 135)		Non-Métis (*n* 1452)	
Characteristic	*n*	%	*n*	%
Sex†				
Male	60	44·4	649	44·7
Female	68	50·4	734	50·5
Other/not specified	7	5·2	69	4·8
Age†				
13	5	3·7	49	3·4
14	97	71·9	1199	82·5
15	31	23·0	194	13·4
16	1	0·7	7	0·5
Not reported	1	0·7	3	0·2

† Self-report.

### Dietary intake

**Figure 1.**
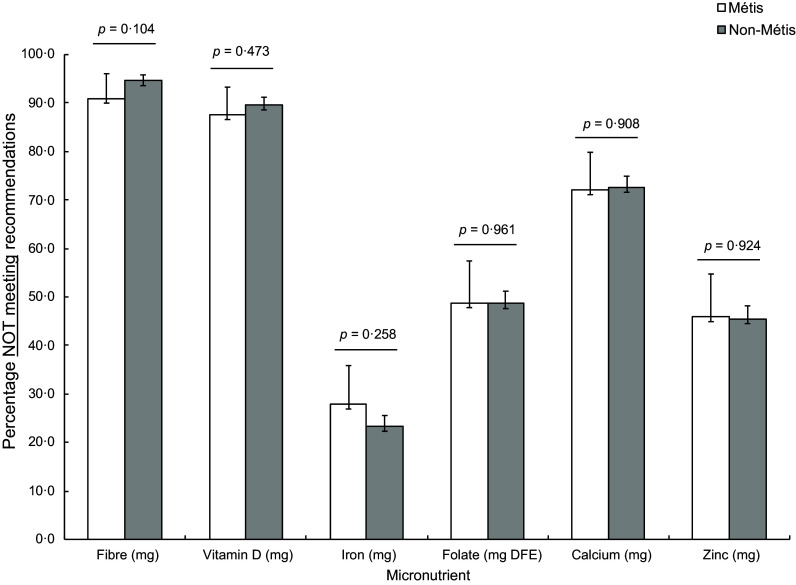
Percentage of participants not meeting recommendations for select nutrients. The estimated average requirement was used for all micronutrients except for fibre (AI). All participants not reporting sex (*n* 72) were excluded for comparison with guidelines that vary by sex (fibre, Fe, Zn). Bars represent the percentage of participants not meeting recommendations; whiskers represent 95 % confidence intervals. *P*-values are for comparisons between Métis and non-Métis participants.

Significant differences in nutrient intakes between Métis and non-Métis participants were observed with higher sugar and saturated fat intake among Métis participants (Table 2), but not for other nutrients. While the percentage of participants not meeting key nutrient recommendations did not differ significantly, most participants were not meeting recommendations for fibre (> 90 %), vitamin D (> 85 %) and Ca (> 70 %) on the day of data collection (Fig. 1). With respect to food group intake, intakes of grain products and meat and alternatives were significantly lower among Métis participants. Significantly more Métis students were not meeting serving requirements for grain products (53·3 %) compared with non-Métis students (41·8 %). Intake of other foods was significantly higher in Métis than non-Métis participants (Table 3).

**Table 2.** Median nutrient intakes and interquartile ranges

Variable	Reference	Recommended Intakes	Métis			Non-Métis			*P*-value
			*n* 129 recalls			*n* 1407 recalls			
			25th	Median	75th	25th	Median	75th	
Energy (kcal)			1372·6	1941·3	2771·3	1387·5	1963·6	2597·5	0·880
Nutrients									
Carbohydrates (g)	EAR	100	169·39	247·3	348·0	175·1	250·5	328·5	0·925
Sugar† (g)			47·2	89·2	153·6	43·7	76·3	113·6	0·014*
Fibre (g)	AI	26§, 38||	9·15	14·8	22·3	9·2	14·3	20·5	0·380
Fat (g)			44·0	68·8	109·4	43·7	68·6	99·6	0·460
Saturated fat (g)			16·1	23·2	41·7	14·4	24·2	36·5	0·270
Monounsaturated fat (g)			13·8	22·7	37·2	13·9	22·5	33·6	0·726
Polyunsaturated fat (g)			6·4	11·7	18·0	7·2	11·7	17·8	0·997
Unsaturated fat (g)	EWCFG	30–45	22·0	35·3	53·7	22·2	34·7	51·3	0·882
Protein‡ (g/kg)	EAR	0·71§, 0·73||	0·76	1·25	1·89	0·91	1·40	2·00	0·152
Vitamin A (µg RAE)	EAR	485§, 630||	206·3	465·7	779·9	222·4	427·5	733·0	0·555
Thiamine, B_1_ (mg)	EAR	0·9§, 1·0||	0·8	1·3	1·8	0·9	1·3	2·0	0·276
Riboflavin, B_2_ (mg)	EAR	0·9§, 1·1||	1·0	1·5	2·5	1·1	1·6	2·4	0·735
Niacin, B_3_ (mg)	EAR	11§, 12||	11·6	17·7	26·1	11·1	17·7	27·0	0·802
Vitamin B_12_ (µg)	EAR	2·0	1·6	3·0	5·6	1·8	3·5	5·6	0·283
Vitamin C (mg)	EAR	56§, 63||	14·0	53·0	146·6	15·8	54·3	133·8	0·804
Vitamin D (µg)	EAR	10	0·5	2·7	5·6	1·1	3·2	6·0	0·131
Folate (µg DFE)	EAR	330	212·0	337·3	526·2	205·0	335·3	503·6	0·702
C (mg)	EAR	1100	457·5	779·0	1144·0	416·5	731·7	1143·0	0·359
Fe (mg)	EAR	7·9§, 7·7||	7·6	11·5	18·5	8·2	12·1	16·9	0·725
Zn (mg)	EAR	7·3§, 8·5||	4·8	8·2	12·5	5·4	8·2	12·2	0·826
Na (mg)	UL	2300	1356·3	2683·2	4302·4	1476·5	2489·2	3762·1	0·244
Saturated fat (% total E)	WHO	< 10 %	9·4	12·4	15·7	8·6	11·6	14·1	0·013*

AI, adequate intake; DFE, dietary folate equivalent; E, energy; EAR, estimated average requirement; EWCFG, Eating Well with Canada’s Food Guide (2007); RAE, retinol activity equivalent; UL, upper limit.

* *P* < 0·05: Mann–Whitney *U* test for differences in median nutrient intakes.

† Sugar includes naturally occurring and added sugars.

‡ Protein requirements based on individual body size; total Métis (*n* 116) and non-Métis (*n* 1173) participants.

§ Recommendations for females.

|| Recommendations for males.

**Table 3.** Eating Well with Canada’s Food Guide (2007) food group servings and percentage not meeting (NM) recommendations

Food group servings	*EWCFG* recommended servings	Métis *n* 129 recalls	Non-Métis *n* 1407 recalls	*P*-value
Grain products	6†, 7‡			
Mean		6·8	7·4	
sd		4·4	4·1	
Median		6·0	7·0	0·039*
IQR		3·5–9·0	4·5–9·7	
NM, *n*		65	561	
NM, %		53·3	41·8	0·014*
Vegetables and fruit	7†, 8‡			
Mean		3·2	3·1	
sd		2·4	2·6	
Median		3·2	2·5	0·183
IQR		1·2–4·7	1·0–4·5	
NM, *n*		115	1259	
NM, %		94·3	93·8	0·844
Milk and alternatives	3–4			
Mean		2·4	2·1	
sd		2·4	1·9	
Median		1·8	1·7	0·433
IQR		0·8–3·2	0·6–3·0	
NM, *n*		95	1047	
NM, %		73·6	74·4	0·848
Meat and alternatives	2†, 3‡			
Mean		2·0	2·4	
sd		1·8	2·0	
Median		1·8	2·0	0·030*
IQR		0·4–3·1	1·0–3·4	
NM, *n*		79	756	
NM, %		64·8	56·3	0·072
Other foods	NA			
Mean		5·2	3·8	
sd		4·7	3·4	
Median		4·0	3·0	< 0·001*
IQR		2·0–7·0	1·0–5·4	
NM, *n*		–	–	
NM, %		–	–	

*EWCFG*, Eating Well with Canada’s Food Guide (2007); IQR, interquartile range; NA, not applicable; NM, not meeting.

* *P* < 0·05: Mann–Whitney *U* for median food group servings and Pearson *χ* ^2^ test for percentage not meeting the recommendation.

† Recommendations for females.

‡ Recommendations for males.

### Food security

A significant difference was observed between the food insecurity status of Métis and non-Métis participants. One-third (33·1 %) of Métis participants were either moderately or severely food insecure, compared with one-fifth (19·1 %) of non-Métis participants (Table 4).

**Table 4.** Food security status of study participants

Food security status	Métis		Non-Métis		*P*-value
	*n* 133		*n* 1401		
	*n*	%	*n*	%	
Food secure	89	66·9	1133	80·9	< 0·001*
Food insecure	44	33·1	268	19·1	

* *P*-value derived from *χ* ^2^ test of association between Métis and non-Métis participants. Bolded value represents statistically significant association (*P* < 0·05).

### Food behaviours and health indicators

The frequency of family dinners and eating breakfast was significantly lower among Métis than non-Métis. Almost twice as many (15·5 %) reported having family dinners 0–1 d/week compared with 8·6 % non-Métis, while more than half (53 %) had breakfast on four or fewer days/week compared with 37·3 % non-Métis (Table 5). Self-reported health was also significantly lower for Métis (49·6 % reporting fair/poor) than non-Métis participants (38·4 %).

**Table 5.** Food behaviours and health indicators of study participants

Food behaviour or health indicator	Total	Métis		Non-Métis		*P*-value
		*n*	%	*n*	%	
Family dinner frequency						
0–1 d/week	136	20	15·5	116	8·6	0·031*
2–4 d/week	215	16	12·4	199	14·7	
5–7 d/week	1134	93	72·1	1041	76·8	
Meal frequency						
Breakfast						
0–4 d/week	591	70	53·0	521	37·3	< 0·001*
5–7 d/week	938	62	47·0	876	62·7	
Lunch						
0–4 d/week	262	29	23·0	233	18·0	0·164
5–7 d/week	1160	97	77·0	1063	82·0	
Previous day meal/snack location						
Breakfast						
Home	1308	108	91·5	1200	90·9	0·823
School/other	130	10	8·5	120	9·1	
Morning snack						
Home	434	38	35·5	396	35·5	0·994
School/other	787	69	64·5	718	64·5	
Lunch						
Home	343	36	28·1	307	22·4	0·144
School/other	1153	92	71·9	1061	77·6	
Afternoon snack						
Home	693	61	56·5	632	54·2	0·656
School/other	580	47	43·5	533	45·8	
Dinner						
Home	1400	121	91·7	1279	91·9	0·910
School/other	123	11	8·3	112	8·1	
Evening snack						
Home	1219	114	95·0	1105	92·8	0·364
School/other	92	6	5·0	86	7·2	
Self-reported health						
Excellent/very good	910	65	50·4	845	61·6	0·013*
Fair/poor	591	64	49·6	527	38·4	

* *P*-value derived from *χ* ^2^ test of association between Métis and non-Métis participants.

The proportion of participants classified as healthy weight and overweight were consistent between Métis and non-Métis participants; however, there were significantly more Métis participants classified as obese (12·6 % *v*. 7·1 %) (Table 6).

**Table 6.** BMI classification of study participants

BMI category (scoring criteria)	Métis		Non-Métis		*P*-value
	*n* 111†		*n* 1094		
	*n*	%	*n*	%	
Healthy weight (−2·0 ≤ z-score ≤ 1·0)	80	72·1	815	74·5	0·582
Overweight (1·0 < z-score ≤ 2·0)	17	15·3	201	18·4	0·418
Obese (z-score > 2·0)	14	12·6	78	7·1	**0·038** *

* Bolded value represents a statistically significant difference between the two proportions, *P* < 0·05 (z-score test for two population proportions).

† *n* 108 due to participants not reporting their height, weight or sex.

## Discussion

### Dietary intakes

While no clear patterns of difference in nutrient or food group intakes were observed between Métis and non-Métis participants, results demonstrate that Manitoba youth overall are consuming low intakes of nutrients and food groups vital for healthy growth and development while consuming high intakes of foods that are recommended to consume in limited amounts. These dietary patterns include inadequate intakes of the vegetables and fruit food group and high intakes of other foods, classified as foods and beverages outside of the 2007 Canada’s Food Guide four food groups (e.g. salty snacks, sweet baked goods, candy, sugar-sweetened beverages)^(12)^.

These findings indicate that Manitoba youth are following dietary patterns, which increase their risk for developing chronic nutrient-related disease in adulthood. The observation that over 25 % of study participants were overweight or obese further adds to the concern that youth are at an increased risk for developing chronic illness later in life, though there is evidence that NCD such as type 2 diabetes are no longer limited to adulthood and are increasing in adolescents^(13)^. In Winnipeg, the largest city in Manitoba, it is projected that 5330 0–19-year-olds will have diagnosed or undiagnosed diabetes by 2032, an increase of 29 % since 2015^(14)^. Notably, in this study, intakes of saturated fat, sugar and other foods were significantly higher among Métis than non-Métis youth. This is cause for alarm as Métis already experience a higher prevalence of type two diabetes and CVD than other Manitobans^(6)^. While consumption of vegetables and fruit was not significantly different among Métis than non-Métis, the inadequate intake of this food group is of note due to previous findings that a much lower percentage of Métis consume vegetables and fruit at least five times per day compared with all other Manitobans (20·9 % *v*. 30·6 %)^(6)^. These findings suggest that there is a risk of this dietary behaviour continuing into adulthood, reinforcing the urgency to develop nutritional interventions focused on youth to promote healthy dietary patterns and reduce the risk of NCD.

### Food security

Food insecurity is defined as having ‘inadequate or insecure access to food due to financial constraints’^(15)^. This study found that 33·1 % of Métis youth were food insecure, which is consistent with the proportion of the Indigenous population in Canada experiencing food insecurity (33·4 %) found by the 2021 Canadian Income Survey^(7)^. However, the Canadian Income Survey used a household-level measure of food insecurity; thus, the results are not directly comparable. Further, Li *et al.* (2022) report on the Indigenous population without differentiating between the three recognised Indigenous groups in Canada. Overall, there is a lack of Métis-specific data on food insecurity status, representing a gap in research. Historical and social factors have contributed to the prevalence of food insecurity among the Indigenous population in Canada. However, these factors have differentially impacted the ways in which each population of Indigenous people experiences food insecurity. The finding that a significantly higher proportion of Métis youth than non-Métis youth are food insecure is cause for concern, especially considering the elevated risk for Métis people to develop diabetes later in life, and warrants a need for more research to further investigate these experiences.

Contributing to food insecurity among Indigenous peoples in Canada is the disruption of traditional food systems and cultural practices due to a history of colonisation^(16)^. Loss of Indigenous control over land and resources, government restrictions around hunting and environmental degradation have all negatively impacted Indigenous peoples’ access to traditional foods^(16)^. Additionally, a history of forced assimilation has disrupted intergenerational knowledge sharing, leading to a loss of traditional knowledge and skills around food for Indigenous peoples^(16)^. The high prevalence of food insecurity among Indigenous peoples indicates a lack of access to both traditional and market foods, which further compounds poor nutrition literacy. All of these intersecting factors must be considered when addressing food insecurity in Indigenous populations.

Data for the FANS study were collected prior to the COVID-19 pandemic, which caused disruptions to employment and community support, leading to changes in food acquisition and distribution patterns^(17)^. Vulnerable groups, including low-income, renters, northern communities, Indigenous and Black Canadians and newcomers, were disproportionately affected by COVID-19^(18)^. This highlights a need to further investigate the impacts of food insecurity in the wake of the pandemic among these groups, including the Métis population.

### Food behaviours and health indicators

Métis participants had fewer family dinners and ate breakfast less frequently than their contemporaries. This may be partly explained by family structure where twice as many (26 %) Métis children live in single-parent families compared with non-Indigenous children (13 %)^(19)^. Food insecurity may be a contributing factor to lower rates of breakfast consumption, as meal skipping, including breakfast, has been observed in food-insecure Canadian young adults^(20)^. These factors could also contribute to the significantly higher rate of obesity observed in Métis participants. These observations, combined with higher rates of NCD in Métis families, may contribute to the higher proportion (half) of Métis participants reporting fair/poor health.

### Limitations and future research

One limitation of this study is the reliance on self-reported Métis identity. Although self-identifying is one element of Métis status, this alone is not accepted by the Manitoba Métis Federation (MMF) nor Métis Nations of other provinces for an individual to be confirmed as Métis. In addition to self-identifying as Métis, the MMF, as the National Government of the Red River Métis, requires that individuals show an ancestral connection to the Historic Métis Community and be accepted by the contemporary Métis Community as a member of the MMF^(21)^. Future research investigating the dietary patterns of Métis youth in Manitoba should be conducted by the MMF with confirmation of Métis Citizenship to reduce potential bias. While the survey was not representative, it is notable that in the 2021 national census (conducted 2 years after data collection), 7·2 % of the provincial population identified as Métis, while Métis students made up 8·5 % of participants in our study^(22)^. Another limitation is the inclusion of a small number (< 4 % of the sample) of 13-year-olds in the cohort. While they have slightly different nutrient requirements, they were included with the rest of the sample, and the proportion was similar across the Metis and non-Metis groups. Therefore, the influence of the inclusion of these individuals would be small.

Many of the analyses conducted in this study were descriptive and measured at the individual level. Our understanding remains limited about the magnitude of impact from structural determinants such as historical and contemporary political contexts, social structures and resource distribution. The structural determinants include factors beyond the control of individuals, including policies, governance and jurisdiction, location, access to appropriate education, housing and culturally safe health and social services, as well as social networks on adolescents’ dietary patterns and lifestyle. At the individual level, access to resources (money, equipment) and knowledge have a strong influence on behaviours. Further discussions with representatives from the Métis government and Indigenous organisations are in progress and critical for the contextualisation and appraisal of these results.

All data in this study were collected as self-reported survey responses. The 24-h dietary recall and FFQ are subject to recall error and inaccurate portion size estimation and do not reflect variations in an individual’s diet day to day as only one 24-h recall was conducted. Reporting bias may also be present in the BMI results as they were also determined from self-reported data. Height is often overestimated, and weight underestimated, potentially leading to underestimated rates of overweight and obesity^(23)^.

The food insecurity section of the survey presented limitations as well. Unlike other food insecurity research conducted in Canada, the study did not collect information about household income, size or parent education level, which are factors contributing to food security status.

### Conclusion

The dietary intakes observed in this study, both among Métis and non-Métis youth, are concerning as many adolescents have dietary patterns that put them at risk for developing health issues in the future. While personal choice is always a factor in food selection, overwhelming evidence suggests youth inhabit food environments that make it nigh impossible to choose a consistently healthy diet^(24)^. This is exacerbated when overlaid by socio-economic and food insecurity^(25)^. As the risk for NCD is increasing among the population, particularly among Métis and other Indigenous youth, there is an urgent need for policy and programming strategies at all levels of government and community to address nutritional shortfalls and food insecurity. The new global policy framework for adolescent nutrition provides excellent guidance in this direction^(26)^, as does this report on Métis food (in)security from British Columbia, Canada^(27)^.

The study data were collected prior to the COVID-19 pandemic, which caused disruptions in employment, community supports and food procurement. The pandemic exacerbated the experience of food insecurity for many vulnerable populations, including the Métis. It also revealed weaknesses in the food system and reinforced the need for long-term and sustainable government programmes and policies to combat food insecurity and support healthy eating across different populations.

Results of this study suggest that Manitoba youth, both Métis and non-Métis, would benefit from culturally relevant school and community-based programmes and policies aimed at promoting healthy diets and supporting healthy dietary patterns long-term. These initiatives paired with strategies to address education, employment and income disparities experienced by the Métis population would contribute to fostering a healthier generation and reducing the risk of nutrition-related chronic disease.
